# MYC drives platinum resistant SCLC that is overcome by the dual PI3K-HDAC inhibitor fimepinostat

**DOI:** 10.1186/s13046-023-02678-1

**Published:** 2023-04-26

**Authors:** Jasmine Chen, Aleks C. Guanizo, W. Samantha N. Jakasekara, Chaitanya Inampudi, Quinton Luong, Daniel J. Garama, Muhammad Alamgeer, Nishant Thakur, Michael DeVeer, Vinod Ganju, D. Neil Watkins, Jason E. Cain, Daniel J. Gough

**Affiliations:** 1 Centre for Cancer Research, Hudson Institute of Medical Research, 27-31 Wright Street, Clayton, Vic 3168 Australia; 2 Department of Molecular and Translational Science, Monash University, 27-31 Wright Street, Clayton, Vic 3168 Australia; 3 Department of Medical Oncology, Monash Health, Clayton, Australia; 4 School of Clinical Sciences, Faculty of Medicine, Nursing and Health Sciences, Monash University, Clayton, Australia; 5 Monash Biomedical Imaging Facility, Monash University, Clayton, Australia; 6 Research Institute in Oncology and Hematology, Cancer Care Manitoba, Winnipeg, MB R3E 0V9 Canada; 7 Department of Internal Medicine, Rady Faculty of Health Sciences, University of Manitoba, Winnipeg, MB R3T 2N2 Canada

**Keywords:** Small cell lung cancer, Platinum resistance, Mouse models, MYC, Fimepinostat

## Abstract

**Background:**

Small cell lung cancer (SCLC) is an aggressive neuroendocrine cancer with an appalling overall survival of less than 5% (Zimmerman et al. J Thor Oncol 14:768-83, 2019). Patients typically respond to front line platinum-based doublet chemotherapy, but almost universally relapse with drug resistant disease. Elevated MYC expression is common in SCLC and has been associated with platinum resistance. This study evaluates the capacity of MYC to drive platinum resistance and through screening identifies a drug capable of reducing MYC expression and overcoming resistance.

**Methods:**

Elevated MYC expression following the acquisition of platinum resistance in vitro and in vivo was assessed. Moreover, the capacity of enforced MYC expression to drive platinum resistance was defined in SCLC cell lines and in a genetically engineered mouse model that expresses MYC specifically in lung tumors. High throughput drug screening was used to identify drugs able to kill MYC-expressing, platinum resistant cell lines. The capacity of this drug to treat SCLC was defined in vivo in both transplant models using cell lines and patient derived xenografts and in combination with platinum and etoposide chemotherapy in an autochthonous mouse model of platinum resistant SCLC.

**Results:**

MYC expression is elevated following the acquisition of platinum resistance and constitutively high MYC expression drives platinum resistance in vitro and in vivo. We show that fimepinostat decreases MYC expression and that it is an effective single agent treatment for SCLC in vitro and in vivo. Indeed, fimepinostat is as effective as platinum-etoposide treatment in vivo. Importantly, when combined with platinum and etoposide, fimepinostat achieves a significant increase in survival.

**Conclusions:**

MYC is a potent driver of platinum resistance in SCLC that is effectively treated with fimepinostat.

**Supplementary Information:**

The online version contains supplementary material available at 10.1186/s13046-023-02678-1.

## Background

Small cell lung cancer (SCLC) is an aggressive neuroendocrine tumor, characterized by a short doubling time, high growth fraction, and early development of widespread metastases [1]. Around two thirds of patients present with extensive stage (ES) disease, defined by spread beyond a tolerable radiation field [2] limiting treatment options to platinum-based chemotherapy. Platinum-based doublet chemotherapy is effective in 60–80% of ES-SCLC, but these responses are short lived [3]. Almost all ES-SCLC patients relapse with drug resistant disease within months for which there is no effective second line therapy. Together this culminates in an appalling overall survival rate of less than 5% [4]. The only substantive change in front-line treatment over the last four decades has been the addition of the immune checkpoint inhibitors Atezolizumab or Durvalumab, however this achieves a modest 2 month increase in overall survival [5, 6] and highlights the need for new and effective drugs to treat SCLC including agents that re-sensitize or prolong the response to platinum-based chemotherapy.

Members of the *MYC* family of oncogenes, *MYCL1*, *MYCN*, and *MYC* are amplified in approximately 20% of SCLCs and are associated with worse survival outcomes [7–10]. *MYC* family amplification is mutually exclusive suggesting broadly overlapping roles in SCLC. There is approximately a threefold increase in the rate of *MYC, MYCN* or *MYCL* amplification in cell lines derived from treated versus untreated patients [11] and a *MYC* transcriptional signature is enriched in tumor biopsy and circulating tumor cell (CTC)-derived SCLC patient derived xenograft (PDX)s from patients with chemoresistant disease [12]. These studies anecdotally link MYC expression to chemoresistance in SCLC. Direct evidence of the role of *MYCL* or *MYCN* in platinum resistance came from a comparison of platinum, etoposide sensitivity in genetically engineered mouse models (GEMM) driven by the loss of *Rb1* and *Trp53* (RP mice) with a model driven by *Rb1* and *Trp53* loss combined with overexpression of *MYCL* or *MYCN* in the epithelium of the adult mouse lung [13]. Furthermore, depleting N-MYC expression through the pharmacological inhibition of the deubiquitinase USP7, that directly deubiquitinates N-MYC and decreases protein stability, restored platinum sensitivity in *MYCN*-overexpressing PDX models [13]. Together, these data provide direct and clear evidence for the role of *MYCN* and *MYCL* expression in platinum resistance and suggest that targeting *MYCN* or *MYCL* expression will restore platinum sensitivity and be a viable therapeutic approach for the treatment of SCLC. However, there is no direct evidence of the role of *MYC* in platinum resistant SCLC, nor drugs identified to extend the duration of platinum therapy response in SCLC patients. This manuscript shows that MYC expression drives platinum resistance in vitro and in vivo and identifies the dual PI3K – HDAC inhibitor fimepinostat as an agent that both reduces MYC expression and kills platinum resistant SCLC cells.

## Materials and methods

### Cell culture

Mouse tumor cell lines were generated following a protocol previously reported [14]. NCI-H146, NCI-H209 and NCI-H69 cells were purchased from the American Type Culture Collection (ATCC, Manassas, VA, USA) and confirmed by short tandem repeat (STR) profiling. SCLC cell lines were cultured in Advanced RPMI supplemented with 1% FCS and 2 mM glutamax in humidified incubators at 37 °C and 5% CO_2_.

### Drug screening

Cells were plated in clear bottom 384 well plates (Greiner bio-one) in culture media using liquid handling robotics (Beckman Coulter, Biomek NXP). Cells were treated with 355 kinase inhibitors (Selleckchem Kinase library, L2000) at a final concentration of 100 nM. Plates were sealed with gas permeable tape (Roll-Seal, Sigma-Aldrich) to stop evaporation and incubated in a humidified culture incubator at 37 °C and 5% CO_2_ for 7 days. Alamar blue viability dye (ThermoFisher) was added using liquid handling robotics and fluorescence measured using a ClarioStar plate reader (BMG Labtech). Background fluorescence was calculated as the mean of wells containing media alone and subtracted from well fluorescence. The mean fluorescence in vehicle control wells was considered 100% viable and fluorescence values for all drug wells expressed as a percentage viability. Z-scores were calculated for each well and a drug considered to be a hit if the Z score was lower than -3. Experiments were performed in duplicate, and a quality control cut off correlation between replicate plates of 0.75 was used. Cell number was optimized for each cell line based on Alamar blue fluorescence after a 7-day incubation.

### Mice

*Rb1*^*fl/fl*^*;Trp53*^*fl/fl*^* and Rb1*^*fl/fl*^*;Trp53*^*fl/fl*^*;Myc*^*LSL/LSL*^ mice have been described previously [15, 16]. NSG mice were purchased from Australian BioResources (Garvan Institute of Medical Research, Sydney, Australia). All animals were housed in a specific-pathogen-free (SPF) vivarium at the Monash Medical Centre (Clayton, Victoria, Australia). All mouse studies were performed in accordance with the ethics approval from the Hudson Institute Animal Ethics Committee. For spontaneous SCLC models, 6–8-week-old mice were anaesthetized with an intraperitoneal injection of 1.25% (v/v) Avertin (Sigma-Aldrich, Missouri, USA) and 10^6^ pfu Ad5-CGRP-Cre (Viral Vector Core Facility, University of Iowa, IA, USA) was delivered intranasally. For xenograft studies, mice were subcutaneously injected with 10^6^ cells resuspended 50% (v/v) Matrigel (Corning, NY, USA). Tumor measurements were taken with digital calipers and tumor volume in mm^3^ calculated as (width^2^ x length)/2. Animals were sacrificed upon reaching ethical endpoints that include, but are not limited to, breathing difficulties, ≥ 20% body weight loss or 800mm^3^ tumor volume. Drug treatments in flank models began when tumors reached 150-200mm^3^. Drug treatment in GEMMs was commenced when tumors were visible by computed tomography. Fimepinostat, 70 mg/kg was administered daily *per. os.* in a 30% captisol solution (CyDex pharmaceuticals). Mice in the platinum/etoposide cohort were injected with carboplatin (60 mg/Kg in PBS) and etoposide (10 mg/kg) intraperitoneally once a week for three weeks with one day between agents. On the day of carboplatin injection mice received an intraperitoneal injection of 1 mL of PBS to minimize kidney toxicity.

### Isolation of primary lung epithelium

Lungs were removed and rinsed in PBS containing MgCl_2_ (100 mg/L) and CaCl_2_ (100 mg/L) and diced. Tissue was digested in collagenase IV (2.5 mg/mL) and DNAse I (100 μg/mL) in PBS at 37 °C for 90 min with rotation. Cells were resuspended in fetal calf serum, passed sequentially through 70 μm and 40 μm filters and plated in DMEM/F12 (Gibco). Cells were allowed to attach for 90 min in a humidified incubator with 5%CO_2_ and media containing epithelial cells was removed and viable lung epithelial cell cells plated for drug treatments.

### Computed tomography

Mice were anaesthetized with isoflurane and Computed Tomography (CT) images were captured using a Siemens Inveon Small Animal PET/SPECT/CT scanner at 39.95 µm resolution, 80 kV, with 500µA current. Mice were fitted with a heart rate and respiration monitor (BioVet) and scans gated to maximum expiration. Images were processed with Fiji and Analyze 12.0 (AnalyzeDirect).

### Western blotting

Cell lysates were resolved through acrylamide gels using SDS-PAGE and transferred to PVDF-FL membranes (Millipore). Membranes were blocked in Odyssey blocking buffer (Li-Cor) and incubated in specific primary antibodies including MYC (Abcam, 32,072), pAkt (Abcam, 38,449), Acetyl-Histone H3 (Abcam, 4729) and actin (Abcam, 3280). Membranes were incubated with IRDye fluorescent-conjugated secondary antibodies (Li-Cor), and protein expression was detected using Odyssey Infrared imaging system (Li-Cor).

### Histology and immunohistochemistry

Lungs were inflation-fixed in 10% neutral buffered formalin for 24 h prior to paraffin embedding. Histological assessment was performed on H&E-stained sections. For immunohistochemical analysis, sections were dewaxed, re-hydrated and subjected to microwave-based antigen retrieval with 20 min boiling in citrate buffer (10 mM citrate, 0.05% Tween-20, pH 6.0) under pressure. Sections were probed with primary antibodies against MYC (Abcam, Cambridge, UK) or PCNA (Dako). Blocking and secondary antibody staining were performed using Vectastain ABC Elite kits (Vector Laboratories, Burlingame, CA, USA).

## Results

### MYC expression drives platinum resistance in vitro and in vivo

**Fig. 1:**
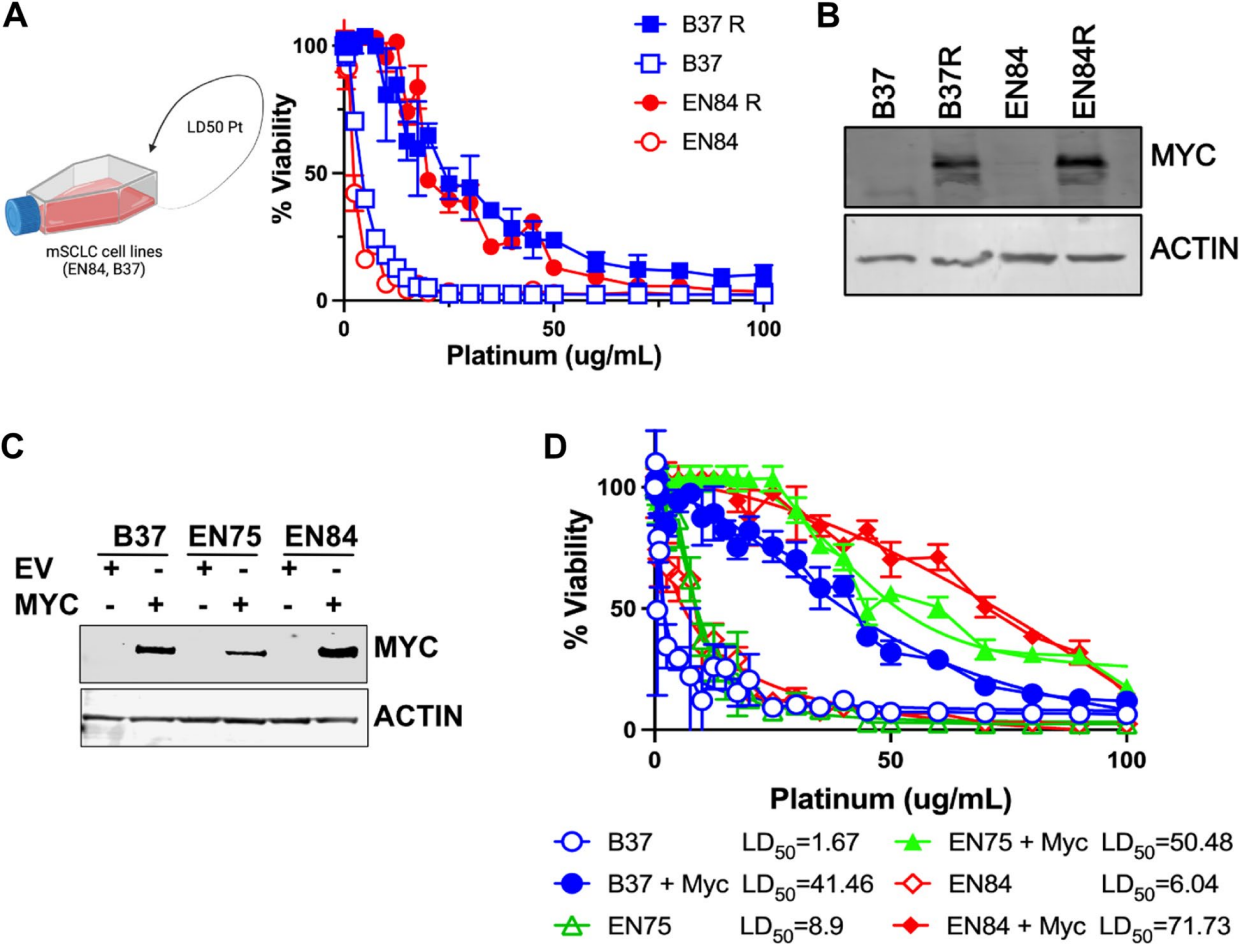
MYC-drives platinum resistant small cell lung cancer in vitro*.* **A** Cell lines derived from the Rb1^−/−^:Trp53^−/−^ mouse model of small cell lung cancer were continuously cultured in an LD50 dose of carboplatin and platinum sensitivity of both platinum naïve and resistant closes determined following treatment with the indicated doses of carboplatin for 7 days. Data is the mean ± SD in three independent experiments. **B** MYC expression in naïve and platinum resistant mouse SCLC cell lines was determined by western blot. Data is representative of 3 independent experiments. **C** Rb1^−/−^:Trp53^−/−^ mouse SCLC cell lines were stably transduced with empty vector (EV) or MYC encoding retrovirus (MYC) and MYC expression confirmed by western blot. Data is representative of 3 independent experiments. **D** The impact of stable MYC overexpression on platinum sensitivity was determined following 7 days exposure to the indicated doses of carboplatin. Data is the mean ± SD in three independent experiments

**Fig. 2:**
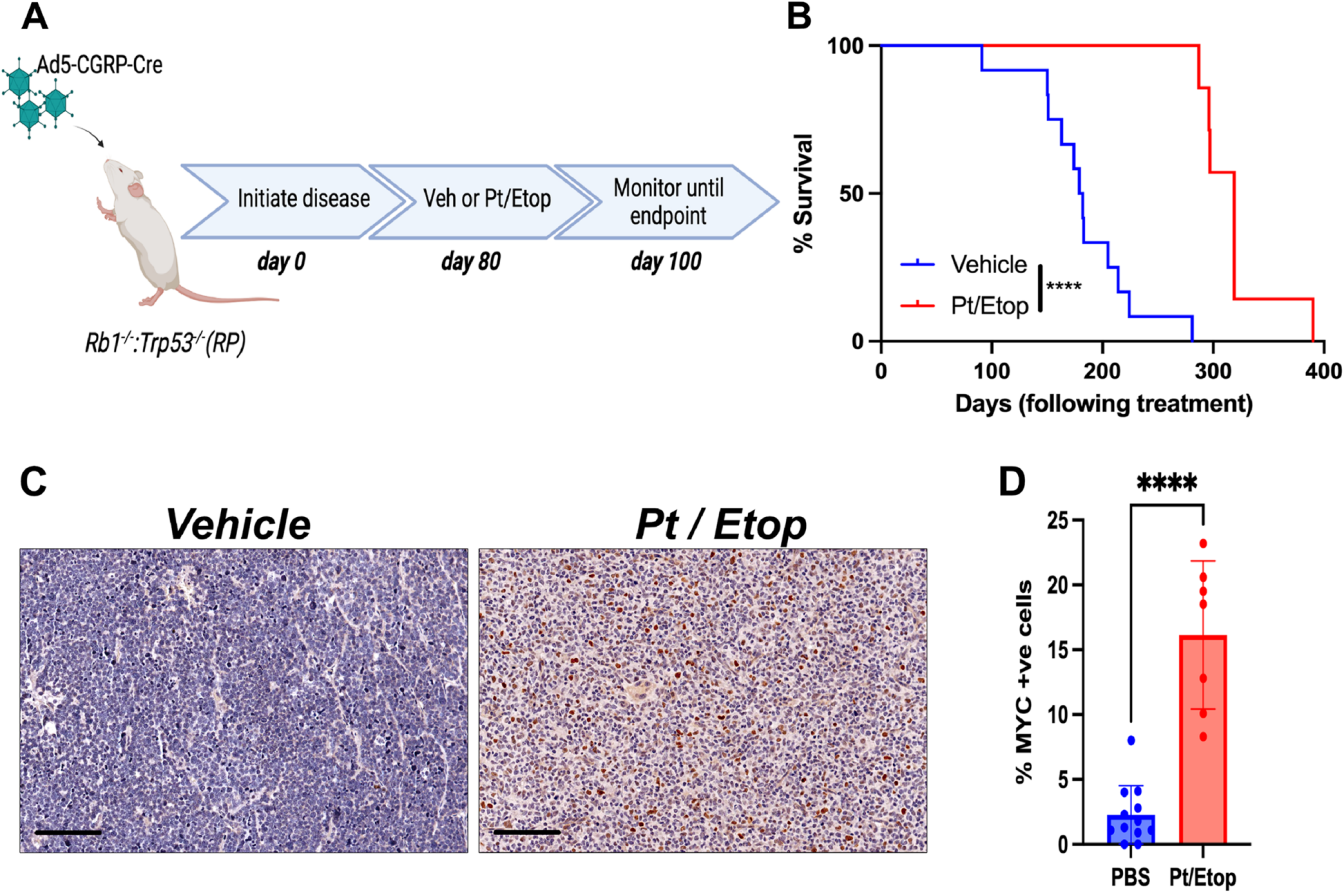
MYC-expression is increased following platinum treatment in vivo*.* **A** Schematic overview of experimental approach. **B** Kaplan–Meier plot showing that carboplatin and etoposide treatment significantly increases the survival in the RP mouse model of SCLC. ****p < 0.0001 Log-rank Mantel-Cox test. **C** representative immunohistochemical analysis of MYC expression in vehicle and carboplatin / etoposide (Pt / etop) (**D**) The % of MYC positive cells per tumor area in each tissue section was determined using the automated positive cell detection feature in QuPath for each animal in each cohort (n > 7 mice per cohort). ****P < 0.0001 students t-test

**Fig. 3:**
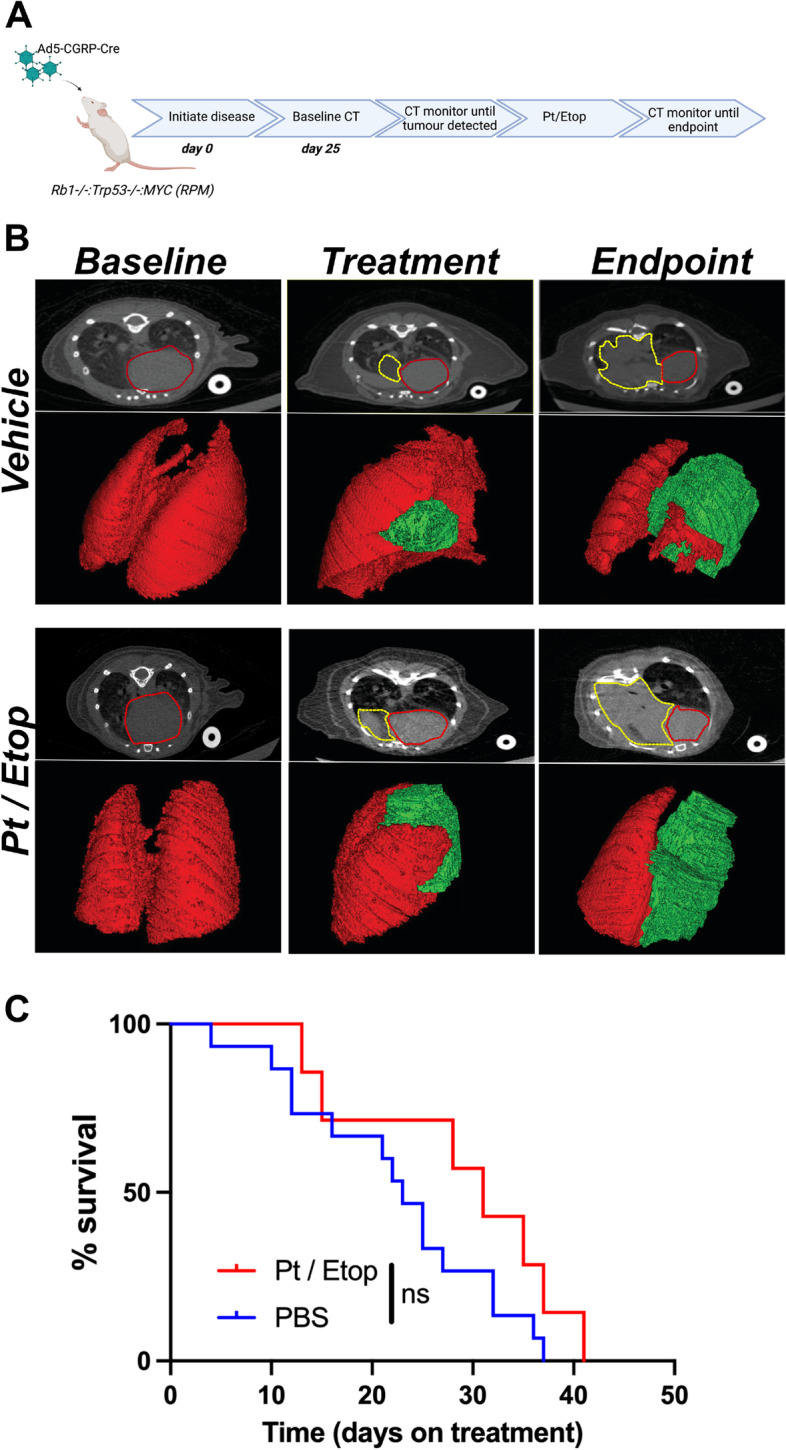
MYC-expression drives platinum resistance in vivo. **A** Schematic overview of experimental approach. **B** Representative CT images of RPM mice at time-points following Cre inhalation but before tumor development (Baseline), when tumor was detected, and treatment initiated (Treatment) and when mice reached ethical endpoint (Endpoint). The area surrounded by the red-dashed line is the heart and the area surrounded by the yellow-dashed line is tumor. CT images were used to generate a 3D reconstruction of the lung. Normal lung is shown in red and tumor in green. Images are representative of at least 8 mice per group. **C** Kaplan–Meier plot showing that carboplatin and etoposide treatment has no significant impact on the survival in the RPM mouse model of SCLC (Log-rank Mantel-Cox test)

The frequency of *MYC* amplification increases in relapsed SCLC [11]. To determine whether *MYC* expression is similarly increased in SCLC cell lines after platinum resistance is acquired two independent cell lines derived from a genetically engineered mouse model driven by the loss of *Rb1* and *Trp53* from lung neuroendocrine cells (RP mouse model) were repeatedly treated with an LD_50_ dose of carboplatin and cells considered resistant if an increase in LD_50_ of at least fivefold was achieved. The LD_50_ of naïve and resistant clones (denoted “R”) was shown to exceed this threshold: B37 4 μg/mL, B37R 24 μg/mL, EN84 2 μg/mL, EN84R 23 μg/mL. Western blot analysis for MYC showed that increased platinum resistance was co-incident with an increase in MYC expression (Fig. 1B). To determine whether MYC expression is associated with platinum treatment in vivo, SCLC was initiated in the RP mouse model by intranasal inoculation of adenoviral Cre-recombinase under control of a neuroendocrine promoter (Ad5-CGRP-Cre) [15, 16]. These mice develop SCLC with a median survival of ~ 200 days. 80 days after disease initiation, mice were randomized into two groups, one received vehicle (PBS) and the other received three cycles of carboplatin (60 mg/kg) and etoposide (10 mg/kg). After three cycles treatment ceased and mice were monitored until they reached ethical endpoint (Fig. 2A). Platinum-etoposide chemotherapy significantly increased the overall survival of RP mice (vehicle 180.5 days, Pt/Etop 319 days, *p* < 0.0001 Log-Rank Mantel-Cox test), but mice did relapse and ultimately succumb to SCLC (Fig. 2B). Immunohistochemical analysis of MYC expression showed very few MYC positive cells in the primary lung tumors from vehicle control mice (mean of 2.28%) but following platinum and etoposide treatment a significant increase in the proportion of MYC positive cells in tumors was observed (mean of 16.14%, *p* < 0.0001, students t-test). Together these data confirm that increased MYC expression is observed following platinum-based chemotherapy and that MYC expression is coincident with acquired platinum resistance in vitro and in vivo. However, these data do not directly assess whether enforced MYC expression drives platinum resistance. To address this, mouse SCLC cell lines derived from RP mice were engineered to stably overexpress MYC and expression confirmed by western blot (Fig. 1C). Three independent matched pairs of cells were treated with titrating concentrations of platinum for 7 days and in each pair of cell lines the overexpression of MYC alone resulted in a significant increase in platinum resistance (Fig. 1D). These data confirm that MYC expression is not only coincident with but is also a driver of platinum resistant SCLC in vitro. To determine whether sustained MYC expression drives platinum resistance in vivo we took advantage of a mouse model of SCLC driven by the loss of *Rb1* and *Trp53* combined with sustained expression of *MYC* exclusively in neuroendocrince cells of the lung (RPM mice) [15]. These mice rapidly develop SCLC and have a median survival of ~ 100 days. Disease was initiated in RPM mice by intranasal inoculation of Ad5-CGRP-Cre and disease onset and progression monitored by computed tomography (CT). A baseline scan was performed 25 days after Cre delivery and mice were subsequently monitored every 10 days by CT (Fig. 3A). When tumor was observed mice were randomized into vehicle and platinum-etoposide treatment groups and treated as described for the RP mouse model. Strikingly, RPM mice were completely refractory to platinum-etoposide treatment. No reduction in tumor volume was observed in RPM mice treated with platinum and etoposide (Fig. 3B). A subtle, but insignificant increase in median survival was observed (vehicle: 23 days on treatment. Platinum and etoposide: 31 days on treatment). Together these data show that elevated MYC expression is not only coincident with platinum resistance but is a potent driver of resistance both in vitro and in vivo.

### Drug screening identifies fimepinostat as a potent inhibitor of platinum resistant SCLC

**Fig. 4:**
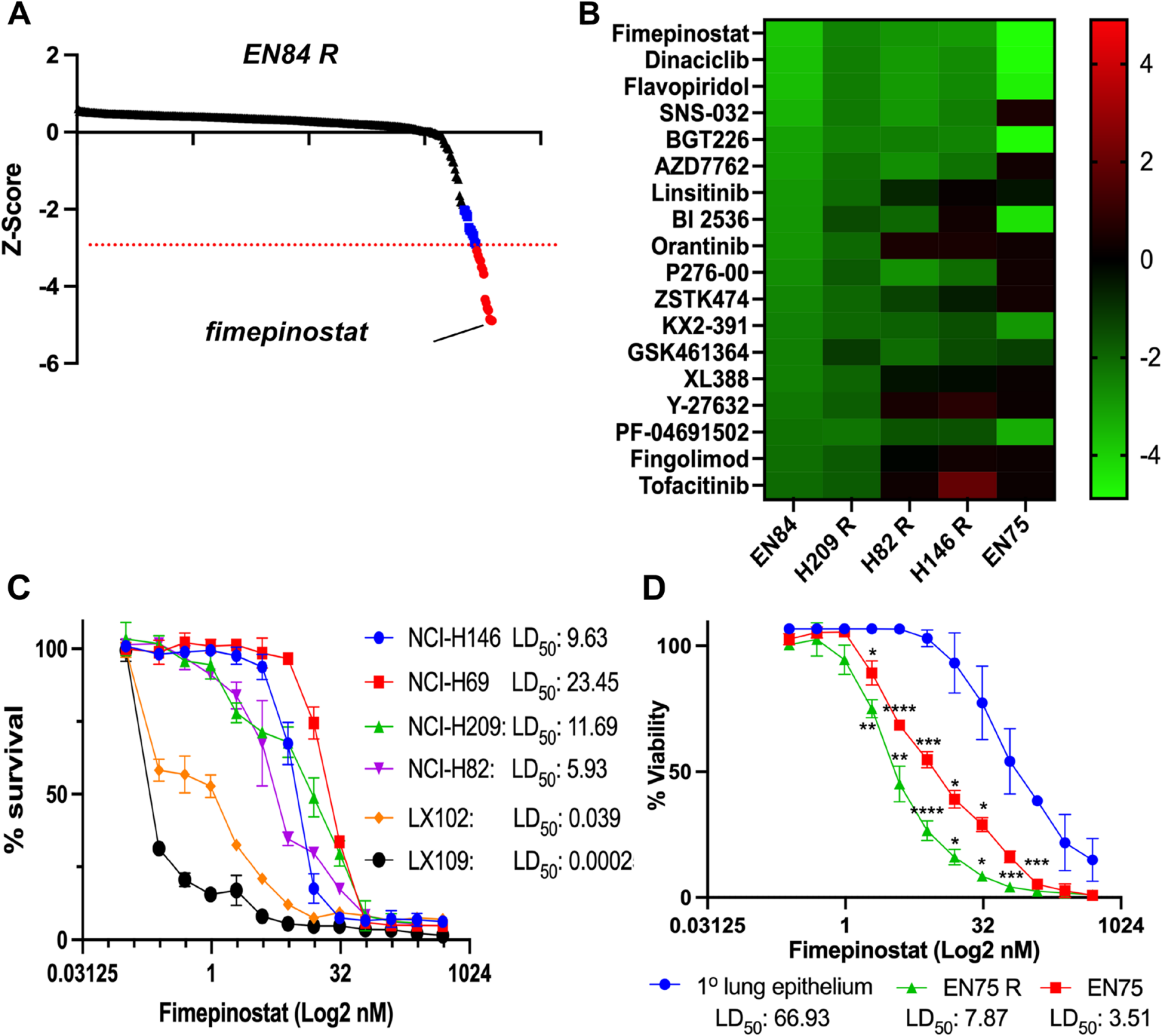
Drug screening identifies fimepinostat as an effective treatment for small cell lung cancer in vitro. **A** Platinum resistant mouse SCLC cell line EN84R was screened for sensitivity to 355 kinase inhibitors at 100 nM for 7 days and the average viability z-score of three independent experiments plotted. Drugs achieving a Z-score of -3 > z > -2 are shown in blue and drugs where z < -3 are shown in red. Fimepinostat is highlighted. **B** Drug screening was performed in triplicate on the indicated cell lines and a heat map of average Z scores for the top 10 hits shown in all cell lines. **C** Dose response curves for fimepinostat sensitivity in a panel of human SCLC cell lines and PDX lines. Data represents the % viability at indicated dose (Log_2_ nM) and LD_50_ (nM) calculated using Prism. **D** Fimepinostat LD_50_ (nM) doses determined for matched platinum naïve and resistant mouse SCLC cell lines and primary (1°) mouse lung epithelium. Statistically significant differences in viability between 1° epithelium and SCLC cell lines were determined by 2-way ANOVA (*p < 0.05, **p < 0.005, ***p < 0.001, ****p < 0.0001)

Identification of drugs that are effective against platinum resistant SCLC, or that will re-sensitize patients to platinum-based therapy are an urgent unmet clinical need. To identify novel drugs, a library of 355 kinase inhibitors (Table S1) was screened for their ability to kill platinum resistant mouse-derived SCLC cell lines at a final concentration of 100 nM. Viability z-scores were calculated in response to each agent and drugs that achieved a z-score less than -3 were prioritized. We identified drugs that have previously been shown to be effective against SCLC including PLK1, Aurora kinase A and CDK inhibitors validating the screening approach. However, the most effective drug was the novel dual PI3K and HDAC inhibitor fimepinostat (Fig. 4A). Importantly, fimepinostat was the most effective drug against all mouse derived cell lines irrespective of their platinum resistance or MYC expression status (Fig. 1B) suggesting that fimepinostat may be broadly effective in the treatment of SCLC. To determine whether fimepinostat efficiently kills human SCLC cell lines we determined the LD_50_ dose of fimepinostat in four established cell lines representative of the recently described neuroendocrine subgroups of SCLC defined by *ASCL1* or *NEUROD1* expression [17] (SCLC-N: NCI-H82. SCLC-A: NCI-H209, NCI-H146 and NCI-H69) and two patient derived xenograft (PDX) lines [18]. Like the observation in mouse derived SCLC cell lines, all human SCLC cell lines were sensitive to fimepinostat with LD_50_ doses between 5.93 and 23.45 nM. This was irrespective of prior chemotherapy treatment, MYC status or SCLC subgroup. The two PDX lines tested were exquisitely sensitive to fimepinostat with LD_50_ values of 0.00025 and 0.039 nM (Fig. 4C). Drugs like PLK1 inhibitors have previously been shown to be effective against SCLC in cell lines but have ultimately failed in clinical testing due to toxicity. Fimepinostat has undergone clinical testing and has been granted orphan drug approval for the treatment of diffuse large B-cell lymphoma [19] and therefore has a known safety profile. However, to indicate whether fimepinostat is likely to have a useful therapeutic window in SCLC the LD_50_ dose was determined for a matched pair of platinum naïve and resistant mouse derived SCLC cell lines and primary mouse lung epithelial cells. We observe significantly less toxicity in normal lung epithelium than observed in either SCLC line (Fig. 4D) suggesting that fimepinostat could be an effective treatment for SCLC whilst preserving normal lung epithelium.

### Fimepinostat reduces MYC expression

MYC is often amplified via gene doubling, tandem duplication, or chromosomal translocation. Indeed, the *MYC* gene is amplified in 40% of human cancers, including breast [20], ovarian [21], prostate [22], hepatocellular [23], colon [24], and lung cancer [25]. MYC has largely been considered undruggable through direct approaches which has led to alternative and indirect approaches to treat MYC expressing tumors. One such approach is the inhibition of bromodomain and extra-terminal domain (BET) family of proteins. BET proteins bind acetylated histone lysine residues, leading to recruitment of P-TEFb via its BRD4 domain to sites of active transcription of genes such as *MYC*. The prototypical BRD2/4 inhibitor, JQ-1 has been shown to decrease MYC expression in other tumor indications [26, 27]. However, we found that treatment of two independent, MYC expressing, platinum resistant mouse derived SCLC cell lines had no impact on MYC expression even at super-physiological doses (Fig. S1).

**Fig. 5:**
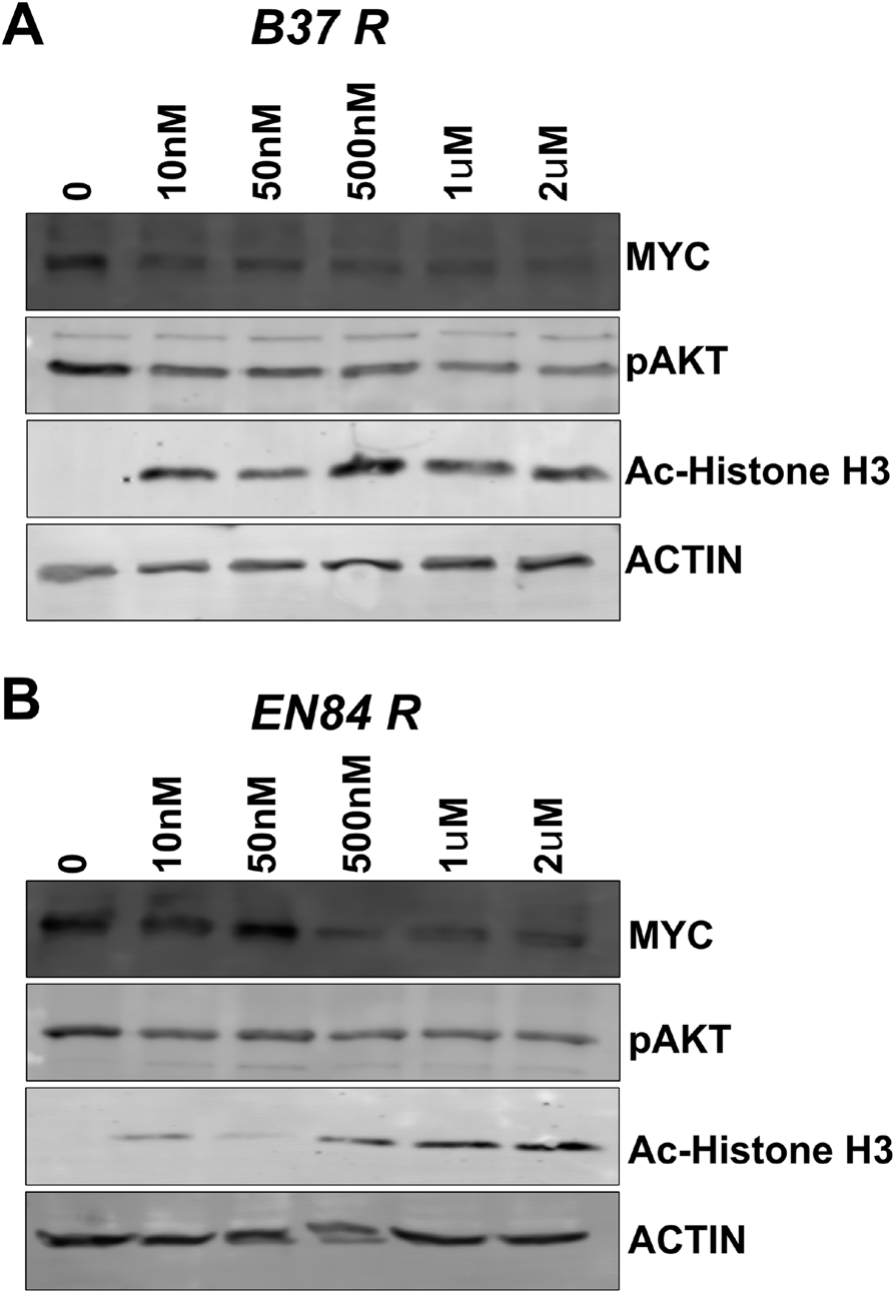
Fimepinostat reduces MYC expression. Western blot analysis showing reduction in MYC expression, phosphorylated (S473) AKT and increased acetylation of histone H3 following a 24-h treatment with the indicated dose of fimepinostat in platinum resistance mouse SCLC cell lines (**A**) B37R and (**B**) EN84R

Fimepinostat is a dual histone deacetylase (HDAC1/HDAC2/HDAC3/HDAC10) and phosphoinositide 3-kinase (PI3Kα/PI3Kβ/PI3Kδ) inhibitor. Both pathways are the focus of intense clinical investigation and have seen agents approved by the FDA for cancer treatment [28, 29]. The PI3K pathway and HDAC activity are implicated in MYC expression. PI3K signaling leads to activation of AKT and inhibition of GSK3β, which phosphorylates MYC at Thr-58 leading to degradation of MYC protein. Hence, inhibition of PI3K prevents inhibition of GSK3β thereby promoting MYC turnover [30–32]. HDAC inhibitors lead to acetylation of MYC at lysine 323 and decreased MYC mRNA and protein expression [33]. Therefore, to determine whether fimepinostat reduced MYC expression in SCLC, two MYC expressing, platinum resistant mouse-derived SCLC cell lines were treated with titrating concentrations of fimepinostat for 24 h. This timepoint precedes the initiation of cell death observed following fimepinostat treatment. Reduction in MYC expression was observed in response to doses as low as 10 nm in B37R2 cells and 500 nm in EN84R2 cells (Fig. 5). This is consistent with studies showing fimepinostat decreases MYC expression in diffuse large B-cell lymphoma and NUT midline carcinoma [34, 35]. Fimepinostat reduced Akt phosphorylation and increased histone H3 acetylation confirming inhibition of PI3K and HDAC respectively (Fig. 5). Importantly, these doses are lower than the drug concentration achieved in patient serum following treatment [36]. Together these data show that fimepinostat is an efficient PI3K and HDAC inhibitor and that it reduces MYC expression. Moreover, we show that fimepinostat efficiently kills SCLC cell lines in vitro and that this is not restricted by MYC expression or platinum resistance status.

### Fimepinostat is an effective SCLC therapy in vivo

**Fig. 6:**
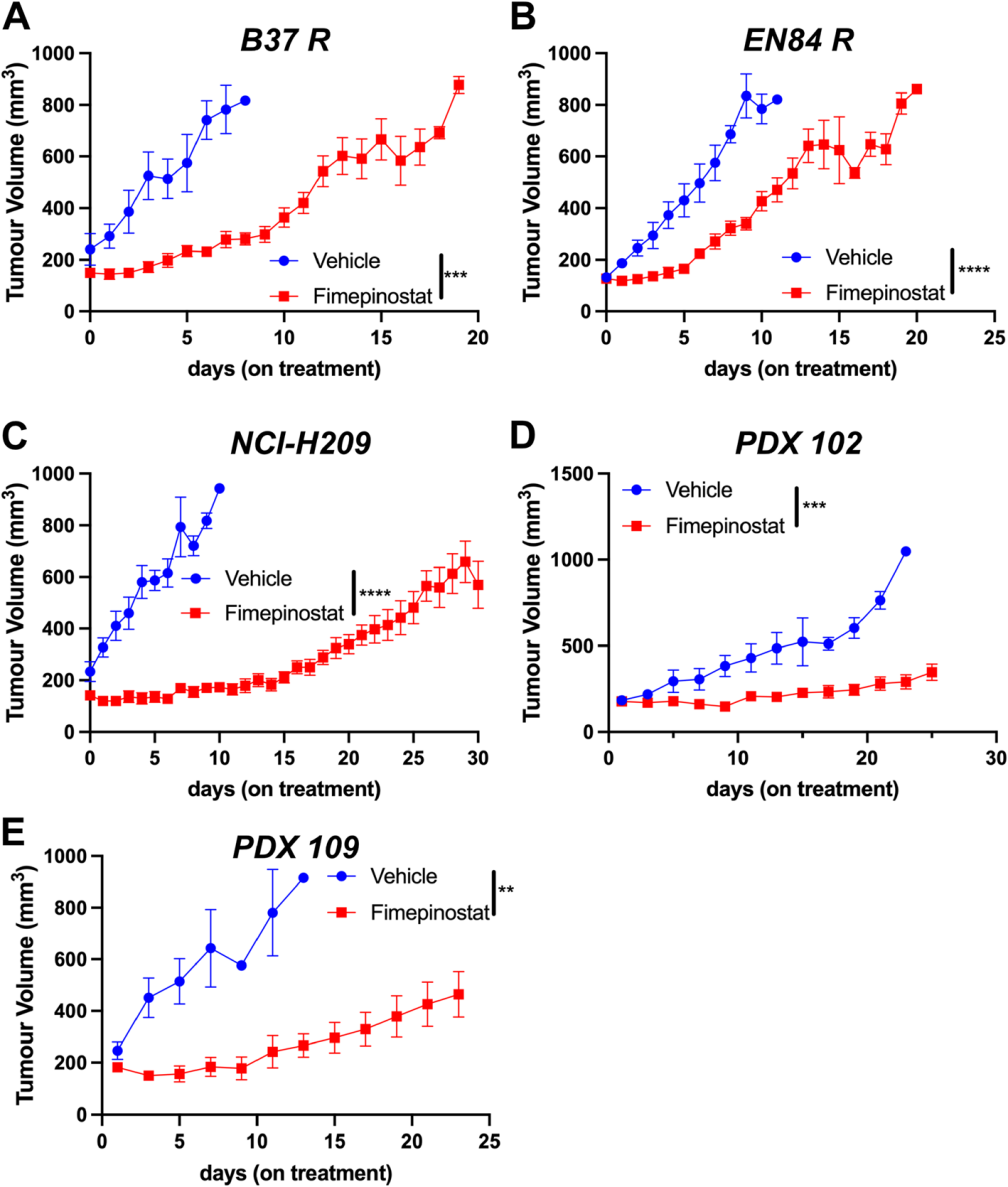
Fimepinostat is an effective SCLC treatment in vivo. Platinum resistant mouse SCLC cell lines (**A**) B37R and (**B**) EN84R, human cell line (**C**) NCI-H209 or PDX cell lines (**D**) PDX102 and (**E**) PDX109 were transplanted subcutaneously into the flanks of recipient mice. When tumors reached 150-200 mm.^3^ mice were treated with fimepinostat (70 mg/Kg, daily, *per os*) and tumor volume measured with calipers. Plotted data is the mean ± SEM for cohorts of at least 8 mice per group. Statistically significant tumor reduction was calculated by paired student’s t-test (**p < 0.005, ***p < 0.001, ****p < 0.0001)

**Fig. 7:**
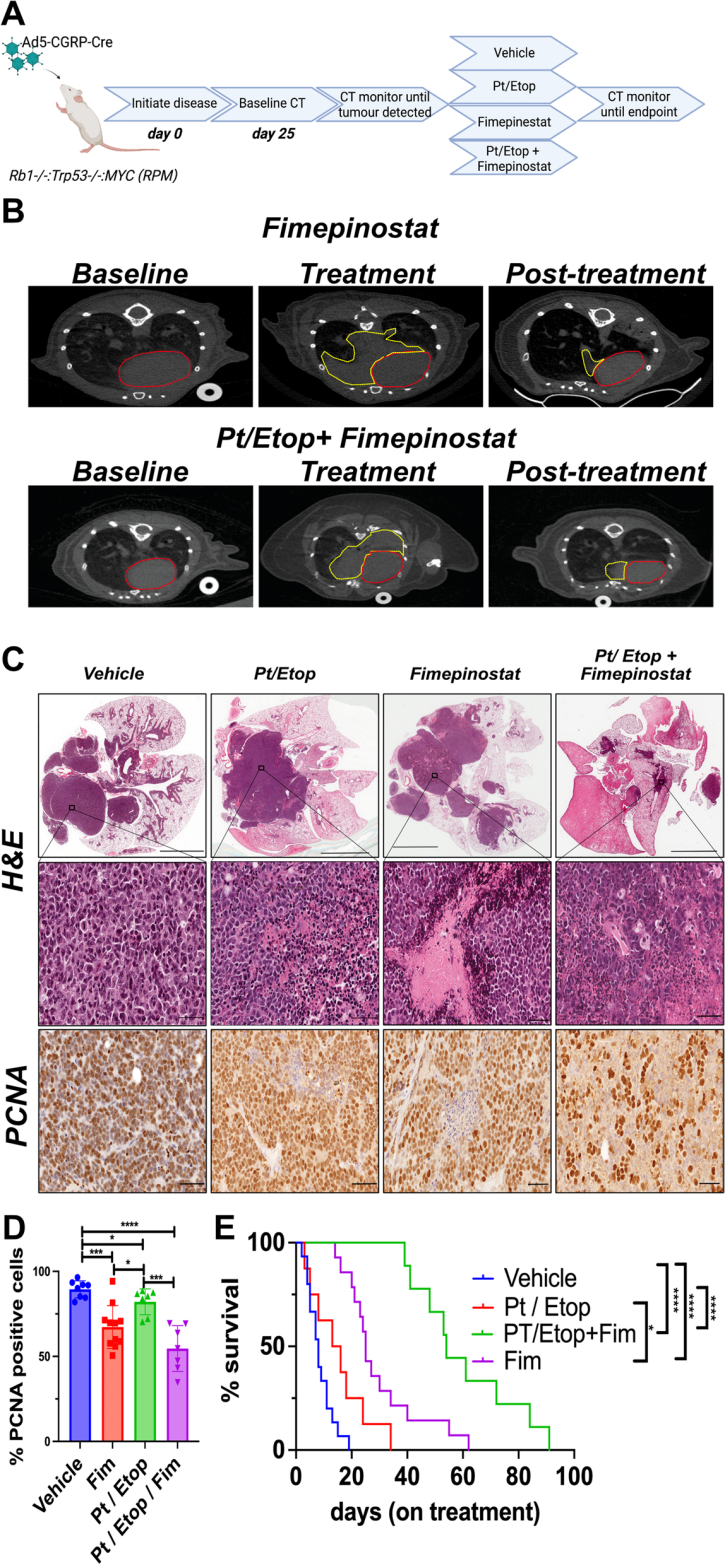
Combination of fimepinostat with standard of care chemotherapy significantly reduces SCLC tumor burden and increases survival in vivo*.* **A** Schematic outline of experimental design (**B**) Representative CT images of RPM mice at time-points following Cre inhalation but before tumor development (Baseline), when tumor was detected, and treatment initiated (Treatment) and when mice reached ethical endpoint (Endpoint). CT images are shown for the fimepinostat alone and fimepinostat/carboplatin/etoposide groups. The area surrounded by the red-dashed line is the heart and the area surrounded by the yellow-dashed line is tumor. Images are representative of at least 8 mice per group. **D** Representative H&E images and immunohistochemical staining for PCNA are shown. Scale bar = 5 mm in whole lung image and 50 μm in 40X magnification. **E** The % of PNCA positive cells per tumor area in each tissue section was determined using the automated positive cell detection feature in QuPath for each animal in each cohort (n > 8 mice per cohort). Data is the mean ± S.D for each treatment group. Statistically significant differences were calculated by students t-test (*p < 0.05, ***p < 0.001, ****p < 0.0001). **F** Kaplan–Meier plot showing that fimepinostat is superior to carboplatin and etoposide treatment and that the combination of fimepinostat (Fim) with carboplatin and etoposide provides a very significant survival advantage in the RPM mouse model of platinum-resistant SCLC (Log-rank Mantel-Cox test, (*p < 0.05, ****p < 0.0001)

Our data show single agent efficacy of fimepinostat at concentrations that are achievable in patients. Therefore, to determine whether fimepinostat can reduce tumor volume in vivo, SCLC cell lines were injected into the flanks of host mice. Mouse derived SCLC cell lines B37R and EN84R were transplanted subcutaneously into the flanks of immune competent C57BL/6 mice. The human SCLC cell line NCI-H209 and PDX lines 102 and 109 were transplanted subcutaneously into the flanks of NOD.Cg-*Prkdc*^*scid*^* Il2rg*^*tm1Wjl*^/SzJ (NSG) mice. Once tumor volume reached 150-200mm^3^ mice received 70 mg/Kg fimepinostat or vehicle by daily oral gavage for 28 days or until tumors reached 800mm^3^ defined as an ethical endpoint. Fimepinostat treatment significantly reduced tumor growth in vivo in all models tested which is remarkable given the rapid growth observed in the vehicle control cohorts (Fig. 6A-E). The previously untreated NCI-H209 and two PDX lines had the most dramatic response to fimepinostat (Fig. 6C-E). Together these data show the single agent efficacy of fimepinostat against tumor cell lines that are representative of the genetic diversity observed in patients. However, these models are very aggressive, are grown in the flank rather than the lung and in the case of the human derived lines are transplanted into immunocompromised mice. Our data show that MYC is a potent driver of platinum resistance (Figs. 1, 2 and 3) and that fimepinostat efficiently reduces MYC expression. Therefore, we hypothesized that whilst fimepinostat efficacy is not dependent on MYC expression it will have the dual capacity to reduce MYC expression extending the duration of response to platinum in addition to its single agent efficacy. To address this, disease was initiated in the autochthonous RPM mouse model driven by deletion of *Trp53*, *Rb1* and gain of *MYC* expression in pulmonary neuroendocrine cells and in the context of an intact immune system. Disease progression was monitored by CT imaging and once tumor was detected mice were randomized into four groups who received (i) vehicle, (ii) carboplatin and etoposide, (iii) fimepinostat or (iv) carboplatin, etoposide and fimepinostat (Fig. 7A). As observed previously no appreciable decrease in tumor size was detected in the platinum / etoposide cohort. In contrast, fimepinostat alone reduced tumor volume (Fig. 7B) and fimepinostat in combination with carboplatin and etoposide led to a very significant reduction in tumor mass (Fig. 7B). Histological and immunohistochemical analysis of lungs showed tumor cell death following each of the treatment arms which was most pronounced in the fimepinostat, carboplatin and etoposide cohort (Fig. 7C). This was accompanied by a significant decrease in cell proliferation based on the percentage of PCNA positive cells within the tumor mass (Fig. 7C, D). Interestingly, in mice treated with fimepinostat, carboplatin and etoposide we observed large regions of disrupted lung tissue architecture likely due to the destruction of tumor that previously occupied the lung (Fig. 7C). No overall survival benefit was observed following carboplatin and etoposide treatment alone. In contrast, fimepinostat monotherapy provides a significant increase in overall survival (*p* < 0.0001, log-rank Mantel-Cox test) and in this model is superior to standard of care chemotherapy. The combination of fimepinostat, carboplatin and etoposide produce a very significant increase in overall survival, taking the median survival from the 8 days on treatment observed in the vehicle group to 54.5 days in the combination therapy group (*p* < 0.0001, log-rank Mantel-Cox test) (Fig. 7E).

Together these data directly show that elevated MYC expression drives platinum resistant SCLC in vitro and in vivo. We identify fimepinostat as a drug that efficiently reduces MYC expression and has single agent efficacy against SCLC in the low nM range. Finally, our data show that the combination of fimepinostat with platinum and etoposide provides a significant survival benefit in an autochthonous mouse model of platinum resistant SCLC.

## Discussion

Small cell lung cancer is an aggressive neuroendocrine tumor with a devastating overall survival rate. Most patients present clinically with extensive-stage SCLC limiting their treatment options to platinum-based doublet chemotherapy. 60–80% of patients initially respond to this chemotherapy regime, however there is nearly universal relapse with platinum resistant disease for which there is currently no effective second line treatment [3, 37]. Amplification of the MYC-family of oncogenes occurs in 20% of SCLC and has been associated with worse prognosis and platinum resistance [7, 8, 10]. An elegant recent study has directly shown that MYCN and MYCL are drivers of platinum resistance in mouse models of SCLC [13]. However, the evidence of a role for MYC in platinum resistant SCLC remains circumstantial, primarily derived from the observation that MYC amplification is more frequent in cell lines derived from platinum resistant patients than treatment naïve lines [10]. In this study we confirm that MYC-overexpression is more frequent following platinum – etoposide chemotherapy in vivo in a mouse model of SCLC and that platinum resistant SCLC cell lines have higher MYC expression than matched platinum naïve cell lines. We provide direct evidence that MYC is a driver of platinum resistance in vitro and in vivo. Cell lines engineered to stably over-express MYC were significantly more resistant to carboplatin than matched control lines. Importantly, we show that an autochthonous mouse model of SCLC driven by the loss of *Trp53*, *Rb1* and gain of *MYC* expression are refractory to platinum-etoposide chemotherapy. In contrast platinum-etoposide treatment of a mouse model driven by the loss of *Trp53*, *Rb1* alone provides a significant survival advantage. Together these data provide direct evidence of the ability of MYC to drive platinum resistance, highlighting the clinical potential to target MYC or MYC-dependent processes to overcome platinum resistant SCLC.

Developing direct MYC inhibitors has been challenging. Instead, we took an unbiased screening approach to identify drugs with single agent efficacy against platinum resistant, MYC expressing SCLC cell lines and identified the dual PI3K-HDAC inhibitor fimepinostat. Fimepinostat has been shown to efficiently reduce MYC protein expression [19], and indeed it was given orphan drug designation for the treatment of relapsed/refractory diffuse B-Cell lymphoma, and has entered phase I clinical testing for pediatric brain tumors with a high incidence of MYC amplification (NCT03893487). We show that fimepinostat efficiently reduces MYC expression in platinum resistant SCLC. Moreover it efficiently kills mouse and human SCLC and PDX lines with a low nanomolar LD_50_ which is in close agreement with an recent independent study in SCLC [38]. It is important to note that in our study we observe that fimepinostat is effective irrespective of the MYC-expression status of the cell line. Together, our demonstrates of the role of MYC in platinum resistant SCLC, the ability of fimepinostat to reduce MYC expression and to have single agent efficacy independent of MYC expression suggests that fimepinostat will be an effective drug for the treatment of SCLC through multiple mechanisms. It is likely to reduce MYC expression in MYC amplified tumors presumably restoring platinum sensitivity in addition to its efficacy in killing platinum naïve and resistant SCLC. Indeed, in an autochthonous mouse model of platinum resistant SCLC we show that fimepinostat significantly increases survival. However, the combination of fimepinostat with standard of care platinum – etoposide chemotherapy provides a greater survival advantage than either agent achieving a durable response that is an increase of ~ 50% of the lifespan of this model.

Histological analysis of lungs following combination fimepinostat, platinum and etoposide treatment showed significant reduction in tumor mass but also revealed damage to the lung architecture. This is likely due to these regions of the lung previously containing tumor that was killed by combination therapy. Due to this, it is possible that the survival benefit observed from combination therapy in our study is an underestimation of what may be observed in patients who receive far more sophisticated clinical care. The data in this manuscript suggests that the addition of fimepinostat to the upfront chemotherapy regime for the treatment of SCLC could improve outcomes and that whilst patients should not necessarily be stratified based on MYC expression those patients with MYC amplification are likely to achieve the benefit of the ability of fimepinostat to reduce MYC expression and prolong platinum response.

## Conclusions

There has been no substantive change in treatment options for platinum resistant small cell lung cancer (SCLC) driving the appalling overall survival rate in these patients. We show that MYC drives platinum resistance in SCLC in vitro and in vivo. Moreover, we identify fimepinostat as a drug capable both of reducing MYC expression and treating SCLC. Importantly, combination treatment with platinum, etoposide and fimepinostat significantly increases survival in an autochthonous mouse model of platinum resistant SCLC. These data together with the known clinical safety profile of fimepinostat provides the rationale to initiate clinical trials of this drug in SCLC patients.

## Supplementary Information

**Additional file 1: Supplemental Table 1.** Drugs screened in this study. **Supplemental Figure 1.** JQ1 has no effect on MYC expression in platinum resistant mouse SCLC cell lines.

## Data Availability

All data is presented in this manuscript. Contact DJGough for requests for material.

## Abbreviations

BET: Bromodomain and extra-terminal domain
CT: Computed tomography
CTC: Circulating tumor cell
GEMM: Genetically engineered mouse model
HDAC: Histone deacetylase
NSG: NOD.Cg-*Prkdc*^*scid*^* Il2rg*^*tm1Wjl*^/SzJ mice
PCNA: Proliferating cell nuclear antigen
PDX: Patient derived xenograft
PI3K: Phosphoinisitide 3-kinase
PLK1: Polo like kinase 1
SCLC: Small cell lung cancer
TUNEL: Terminal deoxynucleotidyl transferase dUTP nick end labeling
