# Supplementary material for: MYC drives platinum resistant SCLC that is overcome by the dual PI3K-HDAC inhibitor fimepinostat
Source: J Exp Clin Cancer Res. 2023 Apr 26;42:100. doi: 10.1186/s13046-023-02678-1 (PMC10131464; doi:10.1186/s13046-023-02678-1)
Supplement: Supplementary file 1 — Additional file 1: Supplemental Table 1. Drugs screened in this study. Supplemental Figure 1. JQ1 has no effect on MYC expression in platinum resistant mouse SCLC cell lines. [file 13046_2023_2678_MOESM1_ESM.docx]

**Supplemental material for:**

***MYC drives platinum resistant SCLC that is overcome by the dual PI3K-HDAC inhibitor fimepinostat.***

Jasmine Chen^1,2^, Aleks C. Guanizo^1,2^, W. Samantha N. Jakasekara^1,2^, Chaitanya Inampudi^1,2^, Quinton Luong^1,2^, Daniel J. Garama^1,2^, Nishant Thakur^1,2^, Michael DeVeer^3^, Vinod Ganju^1,2^, D. Neil Watkins^4,5^, Jason E. Cain^1,2^ and Daniel J. Gough^1,2^

**Table S1.**

| **Drug Name** | **CAS Number** | **Targets** | **Pathway** |
| --- | --- | --- | --- |
| ***Linifanib (ABT-869)*** | 796967-16-3 | CSF-1R,PDGFR,VEGFR | Protein Tyrosine Kinase |
| ***Axitinib*** | 319460-85-0 | c-Kit,VEGFR,PDGFR | Protein Tyrosine Kinase |
| ***Saracatinib (AZD0530)*** | 379231-04-6 | Src,Bcr-Abl | Angiogenesis |
| ***Selumetinib (AZD6244)*** | 606143-52-6 | MEK | MAPK |
| ***BEZ235 (NVP-BEZ235, Dactolisib)*** | 915019-65-7 | PI3K,ATM/ATR,mTOR | PI3K/Akt/mTOR |
| ***Nintedanib (BIBF 1120)*** | 656247-17-5 | VEGFR,PDGFR,FGFR | Protein Tyrosine Kinase |
| ***Afatinib (BIBW2992)*** | 439081-18-2 | EGFR,HER2 | Protein Tyrosine Kinase |
| ***Bosutinib (SKI-606)*** | 380843-75-4 | Src | Angiogenesis |
| ***Cediranib (AZD2171)*** | 288383-20-0 | VEGFR | Protein Tyrosine Kinase |
| ***Dovitinib (TKI-258, CHIR-258)*** | 405169-16-6 | FGFR,FLT3,c-Kit,VEGFR,PDGFR | Angiogenesis |
| ***Canertinib (CI-1033)*** | 267243-28-7 | EGFR,HER2 | Protein Tyrosine Kinase |
| ***PD184352 (CI-1040)*** | 212631-79-3 | MEK | MAPK |
| ***Dasatinib*** | 302962-49-8 | Bcr-Abl,c-Kit,Src | Angiogenesis |
| ***Ridaforolimus (Deforolimus, MK-8669)*** | 572924-54-0 | mTOR | PI3K/Akt/mTOR |
| ***Erlotinib HCl (OSI-744)*** | 183319-69-9 | Autophagy,EGFR | Protein Tyrosine Kinase |
| ***Gefitinib (ZD1839)*** | 184475-35-2 | EGFR | Protein Tyrosine Kinase |
| ***Imatinib Mesylate (STI571)*** | 220127-57-1 | c-Kit,Bcr-Abl,PDGFR | Protein Tyrosine Kinase |
| ***Lapatinib (GW-572016) Ditosylate*** | 388082-77-7 | HER2,EGFR | Protein Tyrosine Kinase |
| ***Motesanib Diphosphate (AMG-706)*** | 857876-30-3 | VEGFR,PDGFR,c-Kit | Protein Tyrosine Kinase |
| ***Nilotinib (AMN-107)*** | 641571-10-0 | Bcr-Abl | Angiogenesis |
| ***NVP-AEW541*** | 475489-16-8 | IGF-1R | Protein Tyrosine Kinase |
| ***Pazopanib HCl (GW786034 HCl)*** | 635702-64-6 | VEGFR,PDGFR,c-Kit | Protein Tyrosine Kinase |
| ***PD0325901*** | 391210-10-9 | MEK | MAPK |
| ***PI-103*** | 371935-74-9 | PI3K,Autophagy,DNA-PK,mTOR | PI3K/Akt/mTOR |
| ***Rapamycin (Sirolimus)*** | 53123-88-9 | Autophagy,mTOR | PI3K/Akt/mTOR |
| ***Sorafenib Tosylate*** | 475207-59-1 | PDGFR,Raf,VEGFR | MAPK |
| ***Sunitinib Malate*** | 341031-54-7 | VEGFR,PDGFR,c-Kit | Protein Tyrosine Kinase |
| ***Tandutinib (MLN518)*** | 387867-13-2 | FLT3 | Angiogenesis |
| ***Temsirolimus (CCI-779, NSC 683864)*** | 162635-04-3 | mTOR | PI3K/Akt/mTOR |
| ***Vandetanib (ZD6474)*** | 443913-73-3 | VEGFR | Protein Tyrosine Kinase |
| ***VX-680 (Tozasertib, MK-0457)*** | 639089-54-6 | Aurora Kinase | Cell Cycle |
| ***Y-27632 2HCl*** | 129830-38-2 | Autophagy,ROCK | Cell Cycle |
| ***Enzastaurin (LY317615)*** | 170364-57-5 | PKC | TGF-beta/Smad |
| ***AC480 (BMS-599626)*** | 714971-09-2 | HER2,EGFR | Protein Tyrosine Kinase |
| ***Masitinib (AB1010)*** | 790299-79-5 | PDGFR,c-Kit | Protein Tyrosine Kinase |
| ***GDC-0941*** | 957054-30-7 | PI3K | PI3K/Akt/mTOR |
| ***SL-327*** | 305350-87-2 | MEK | MAPK |
| ***Crizotinib (PF-02341066)*** | 877399-52-5 | c-Met,ALK | Protein Tyrosine Kinase |
| ***PHA-665752*** | 477575-56-7 | c-Met | Protein Tyrosine Kinase |
| ***ZSTK474*** | 475110-96-4 | PI3K | PI3K/Akt/mTOR |
| ***SB216763*** | 280744-09-4 | GSK-3 | PI3K/Akt/mTOR |
| ***SB203580*** | 152121-47-6 | p38 MAPK | MAPK |
| ***SB202190 (FHPI)*** | 152121-30-7 | p38 MAPK | MAPK |
| ***MK-2206 2HCl*** | 1032350-13-2 | Akt | PI3K/Akt/mTOR |
| ***SU11274*** | 658084-23-2 | c-Met | Protein Tyrosine Kinase |
| ***Brivanib (BMS-540215)*** | 649735-46-6 | FGFR,VEGFR | Protein Tyrosine Kinase |
| ***NVP-ADW742*** | 475488-23-4 | IGF-1R | Protein Tyrosine Kinase |
| ***OSI-906 (Linsitinib)*** | 867160-71-2 | IGF-1R | Protein Tyrosine Kinase |
| ***KU-55933 (ATM Kinase Inhibitor)*** | 587871-26-9 | ATM/ATR | DNA Damage |
| ***GSK1904529A*** | 1089283-49-7 | IGF-1R | Protein Tyrosine Kinase |
| ***PF-04217903*** | 956905-27-4 | c-Met | Protein Tyrosine Kinase |
| ***MLN8054*** | 869363-13-3 | Aurora Kinase | Cell Cycle |
| ***Vatalanib (PTK787) 2HCl*** | 212141-51-0 | c-Kit,VEGFR | Protein Tyrosine Kinase |
| ***U0126-EtOH*** | 1173097-76-1 | MEK | MAPK |
| ***ZM 447439*** | 331771-20-1 | Aurora Kinase | Cell Cycle |
| ***GDC-0879*** | 905281-76-7 | Raf | MAPK |
| ***LY294002*** | 154447-36-6 | Autophagy,PI3K | PI3K/Akt/mTOR |
| ***OSU-03012 (AR-12)*** | 742112-33-0 | PDK-1 | PI3K/Akt/mTOR |
| ***Danusertib (PHA-739358)*** | 827318-97-8 | c-RET,FGFR,Bcr-Abl,Aurora Kinase | Cell Cycle |
| ***TAE684 (NVP-TAE684)*** | 761439-42-3 | ALK | Protein Tyrosine Kinase |
| ***BI 2536*** | 755038-02-9 | PLK | Cell Cycle |
| ***Foretinib (GSK1363089)*** | 849217-64-7 | VEGFR,c-Met | Protein Tyrosine Kinase |
| ***SGX-523*** | 1022150-57-7 | c-Met | Protein Tyrosine Kinase |
| ***GSK690693*** | 937174-76-0 | Akt | PI3K/Akt/mTOR |
| ***JNJ-38877605*** | 943540-75-8 | c-Met | Protein Tyrosine Kinase |
| ***Palbociclib (PD-0332991) HCl*** | 827022-32-2 | CDK | Cell Cycle |
| ***Triciribine*** | 35943-35-2 | Akt | PI3K/Akt/mTOR |
| ***XL147*** | 956958-53-5 | PI3K | PI3K/Akt/mTOR |
| ***Cabozantinib (XL184, BMS-907351)*** | 849217-68-1 | FLT3,Tie-2,c-Kit,c-Met,VEGFR,Axl | Protein Tyrosine Kinase |
| ***Everolimus (RAD001)*** | 159351-69-6 | mTOR | PI3K/Akt/mTOR |
| ***BMS-754807*** | 1001350-96-4 | IGF-1R,Trk receptor,c-Met | Protein Tyrosine Kinase |
| ***Alisertib (MLN8237)*** | 1028486-01-2 | Aurora Kinase | Cell Cycle |
| ***AT9283*** | 896466-04-9 | JAK,Aurora Kinase,Bcr-Abl | JAK/STAT |
| ***Brivanib Alaninate (BMS-582664)*** | 649735-63-7 | VEGFR,FGFR | Protein Tyrosine Kinase |
| ***AG-490 (Tyrphostin B42)*** | 133550-30-8 | JAK,EGFR | Protein Tyrosine Kinase |
| ***SNS-032 (BMS-387032)*** | 345627-80-7 | CDK | Cell Cycle |
| ***Barasertib (AZD1152-HQPA)*** | 722544-51-6 | Aurora Kinase | Cell Cycle |
| ***PLX-4720*** | 918505-84-7 | Raf | MAPK |
| ***Roscovitine (Seliciclib,CYC202)*** | 186692-46-6 | CDK | Cell Cycle |
| ***SNS-314 Mesylate*** | 1146618-41-8 | Aurora Kinase | Cell Cycle |
| ***Lenvatinib (E7080)*** | 417716-92-8 | VEGFR | Protein Tyrosine Kinase |
| ***CP-724714*** | 537705-08-1 | EGFR,HER2 | Protein Tyrosine Kinase |
| ***TGX-221*** | 663619-89-4 | PI3K | PI3K/Akt/mTOR |
| ***WZ3146*** | 1214265-56-1 | EGFR | Protein Tyrosine Kinase |
| ***CYC116*** | 693228-63-6 | Aurora Kinase,VEGFR | Cell Cycle |
| ***WZ4002*** | 1213269-23-8 | EGFR | Protein Tyrosine Kinase |
| ***PD98059*** | 167869-21-8 | MEK | MAPK |
| ***Regorafenib (BAY 73-4506)*** | 755037-03-7 | c-RET,VEGFR | Protein Tyrosine Kinase |
| ***WZ8040*** | 1214265-57-2 | EGFR | Protein Tyrosine Kinase |
| ***ENMD-2076*** | 934353-76-1 | Aurora Kinase,FLT3,VEGFR | Angiogenesis |
| ***CUDC-101*** | 1012054-59-9 | HDAC,HER2,EGFR | Epigenetics |
| ***PIK-75*** | 372196-77-5 | PI3K,DNA-PK | PI3K/Akt/mTOR |
| ***Tivozanib (AV-951)*** | 475108-18-0 | VEGFR,PDGFR,c-Kit | Protein Tyrosine Kinase |
| ***YM201636*** | 371942-69-7 | PI3K | PI3K/Akt/mTOR |
| ***OSI-930*** | 728033-96-3 | c-Kit,CSF-1R,VEGFR | Protein Tyrosine Kinase |
| ***KU-0063794*** | 938440-64-3 | mTOR | PI3K/Akt/mTOR |
| ***AG-1024*** | 65678-07-1 | IGF-1R | Protein Tyrosine Kinase |
| ***Amuvatinib (MP-470)*** | 850879-09-3 | FLT3,c-RET,PDGFR,c-Kit | Protein Tyrosine Kinase |
| ***JNJ-7706621*** | 443797-96-4 | CDK,Aurora Kinase | Cell Cycle |
| ***PD173074*** | 219580-11-7 | VEGFR,FGFR | Angiogenesis |
| ***WYE-354*** | 1062169-56-5 | mTOR | PI3K/Akt/mTOR |
| ***Vemurafenib (PLX4032, RG7204)*** | 918504-65-1 | Raf | MAPK |
| ***BX-795*** | 702675-74-9 | IκB/IKK,PDK-1 | PI3K/Akt/mTOR |
| ***BX-912*** | 702674-56-4 | PDK-1 | PI3K/Akt/mTOR |
| ***Zoledronic Acid*** | 118072-93-8 | Rac | Cell Cycle |
| ***Genistein*** | 446-72-0 | Topoisomerase,EGFR | Protein Tyrosine Kinase |
| ***TG100-115*** | 677297-51-7 | PI3K | PI3K/Akt/mTOR |
| ***GSK1059615*** | 958852-01-2 | PI3K,mTOR | PI3K/Akt/mTOR |
| ***MGCD-265*** | 875337-44-3 | Tie-2,VEGFR,c-Met | Protein Tyrosine Kinase |
| ***Rigosertib (ON-01910)*** | 1225497-78-8 | PLK | Cell Cycle |
| ***Ki8751*** | 228559-41-9 | PDGFR,c-Kit,VEGFR | Protein Tyrosine Kinase |
| ***Ruxolitinib (INCB018424)*** | 941678-49-5 | JAK | JAK/STAT |
| ***Pelitinib (EKB-569)*** | 257933-82-7 | EGFR | Protein Tyrosine Kinase |
| ***Aurora A Inhibitor I*** | 1158838-45-9 | Aurora Kinase | Cell Cycle |
| ***PHA-680632*** | 398493-79-3 | Aurora Kinase | Cell Cycle |
| ***VX-745*** | 209410-46-8 | p38 MAPK | MAPK |
| ***Thiazovivin*** | 1226056-71-8 | ROCK | Cell Cycle |
| ***SP600125*** | 129-56-6 | JNK | MAPK |
| ***AZD6482*** | 1173900-33-8 | PI3K | PI3K/Akt/mTOR |
| ***TSU-68 (SU6668, Orantinib)*** | 252916-29-3 | VEGFR,PDGFR,FGFR | Protein Tyrosine Kinase |
| ***GSK429286A*** | 864082-47-3 | ROCK | Cell Cycle |
| ***Pimasertib (AS-703026)*** | 1236699-92-5 | MEK | MAPK |
| ***HMN-214*** | 173529-46-9 | PLK | Cell Cycle |
| ***AEE788 (NVP-AEE788)*** | 497839-62-0 | HER2,VEGFR,EGFR | Protein Tyrosine Kinase |
| ***PHA-793887*** | 718630-59-2 | CDK | Cell Cycle |
| ***PIK-93*** | 593960-11-3 | PI3K | PI3K/Akt/mTOR |
| ***Ponatinib (AP24534)*** | 943319-70-8 | PDGFR,FGFR,VEGFR,Bcr-Abl | Angiogenesis |
| ***LY2228820*** | 862507-23-1 | p38 MAPK | MAPK |
| ***CCT129202*** | 942947-93-5 | Aurora Kinase | Cell Cycle |
| ***SAR245409 (XL765)*** | 1349796-36-6 | PI3K,mTOR | PI3K/Akt/mTOR |
| ***AT7519*** | 844442-38-2 | CDK | Cell Cycle |
| ***Quizartinib (AC220)*** | 950769-58-1 | FLT3 | Angiogenesis |
| ***Hesperadin*** | 422513-13-1 | Aurora Kinase | Cell Cycle |
| ***BIX 02188*** | 1094614-84-2 | MEK | MAPK |
| ***BIX 02189*** | 1094614-85-3 | MEK | MAPK |
| ***AZD7762*** | 860352-01-8 | Chk | Cell Cycle |
| ***R406 (free base)*** | 841290-80-0 | Syk | Angiogenesis |
| ***CP-673451*** | 343787-29-1 | PDGFR | Protein Tyrosine Kinase |
| ***AZD8055*** | 1009298-09-2 | mTOR | PI3K/Akt/mTOR |
| ***PHT-427*** | 1191951-57-1 | PDK-1,Akt | PI3K/Akt/mTOR |
| ***KRN 633*** | 286370-15-8 | PDGFR,VEGFR | Protein Tyrosine Kinase |
| ***AT7867*** | 857531-00-1 | S6 Kinase,Akt | PI3K/Akt/mTOR |
| ***BMS-777607*** | 1025720-94-8 | Axl,c-Met | Protein Tyrosine Kinase |
| ***PD318088*** | 391210-00-7 | MEK | MAPK |
| ***KU-60019*** | 925701-49-1 | ATM/ATR | DNA Damage |
| ***BS-181 HCl*** | 1397219-81-6 | CDK | Cell Cycle |
| ***Fasudil (HA-1077) HCl*** | 105628-07-7 | ROCK,Autophagy | Cell Cycle |
| ***BIRB 796 (Doramapimod)*** | 285983-48-4 | p38 MAPK | MAPK |
| ***Tie2 kinase inhibitor*** | 948557-43-5 | Tie-2 | Protein Tyrosine Kinase |
| ***H 89 2HCl*** | 130964-39-5 | PKA | PI3K/Akt/mTOR |
| ***TWS119*** | 601514-19-6 | GSK-3 | PI3K/Akt/mTOR |
| ***Acadesine*** | 2627-69-2 | AMPK | PI3K/Akt/mTOR |
| ***PF-573228*** | 869288-64-2 | FAK | Angiogenesis |
| ***BMS-265246*** | 582315-72-8 | CDK | Cell Cycle |
| ***AZD8330*** | 869357-68-6 | MEK | MAPK |
| ***Neratinib (HKI-272)*** | 698387-09-6 | HER2,EGFR | Protein Tyrosine Kinase |
| ***KW-2449*** | 1000669-72-6 | Aurora Kinase,Bcr-Abl,FLT3 | Angiogenesis |
| ***RAF265 (CHIR-265)*** | 927880-90-8 | VEGFR,Raf | MAPK |
| ***PF-4708671*** | 1255517-76-0 | S6 Kinase | PI3K/Akt/mTOR |
| ***LY2784544*** | 1229236-86-5 | JAK | JAK/STAT |
| ***BGJ398 (NVP-BGJ398)*** | 872511-34-7 | FGFR | Angiogenesis |
| ***AST-1306*** | 1050500-29-2 | EGFR | Protein Tyrosine Kinase |
| ***AZD8931 (Sapitinib)*** | 848942-61-0 | HER2,EGFR | Protein Tyrosine Kinase |
| ***GSK461364*** | 929095-18-1 | PLK | Cell Cycle |
| ***R406*** | 841290-81-1 | Syk,FLT3 | Angiogenesis |
| ***SGI-1776 free base*** | 1025065-69-3 | Pim | JAK/STAT |
| ***BMS-794833*** | 1174046-72-0 | VEGFR,c-Met | Protein Tyrosine Kinase |
| ***NVP-BHG712*** | 940310-85-0 | Raf,Src,Bcr-Abl,VEGFR,Ephrin receptor | Protein Tyrosine Kinase |
| ***OSI-420*** | 183320-51-6 | EGFR | Protein Tyrosine Kinase |
| ***PIK-293*** | 900185-01-5 | PI3K | PI3K/Akt/mTOR |
| ***AZ 960*** | 905586-69-8 | JAK | JAK/STAT |
| ***Mubritinib (TAK 165)*** | 366017-09-6 | HER2 | Protein Tyrosine Kinase |
| ***PP242*** | 1092351-67-1 | mTOR,Autophagy | PI3K/Akt/mTOR |
| ***CYT387*** | 1056634-68-4 | JAK | JAK/STAT |
| ***SB590885*** | 405554-55-4 | Raf | MAPK |
| ***Apatinib*** | 811803-05-1 | VEGFR | Protein Tyrosine Kinase |
| ***CAL-101 (Idelalisib, GS-1101)*** | 870281-82-6 | PI3K | PI3K/Akt/mTOR |
| ***PIK-294*** | 900185-02-6 | PI3K | PI3K/Akt/mTOR |
| ***Telatinib*** | 332012-40-5 | VEGFR,PDGFR,c-Kit | Protein Tyrosine Kinase |
| ***Volasertib (BI 6727)*** | 755038-65-4 | PLK | Cell Cycle |
| ***Palomid 529 (P529)*** | 914913-88-5 | mTOR | PI3K/Akt/mTOR |
| ***Degrasyn (WP1130)*** | 856243-80-6 | DUB,Bcr-Abl | Angiogenesis |
| ***BKM120 (NVP-BKM120, Buparlisib)*** | 944396-07-0 | PI3K | PI3K/Akt/mTOR |
| ***Asiatic Acid*** | 464-92-6 | p38 MAPK | MAPK |
| ***Honokiol*** | 35354-74-6 | MEK,Akt | PI3K/Akt/mTOR |
| ***Indirubin*** | 479-41-4 | GSK-3 | PI3K/Akt/mTOR |
| ***Quercetin*** | 117-39-5 | PKC,Src,PI3K,Sirtuin | Epigenetics |
| ***Chrysophanic Acid*** | 481-74-3 | mTOR,EGFR | Protein Tyrosine Kinase |
| ***Imatinib (STI571)*** | 152459-95-5 | PDGFR | Protein Tyrosine Kinase |
| ***Phenformin HCl*** | 834-28-6 | AMPK | PI3K/Akt/mTOR |
| ***TAK-733*** | 1035555-63-5 | MEK | MAPK |
| ***AZD5438*** | 602306-29-6 | CDK | Cell Cycle |
| ***PP121*** | 1092788-83-4 | DNA-PK,PDGFR,mTOR | Protein Tyrosine Kinase |
| ***OSI-027*** | 936890-98-1 | mTOR | PI3K/Akt/mTOR |
| ***Fostamatinib (R788)*** | 901119-35-5 | Syk | Angiogenesis |
| ***LY2603618*** | 911222-45-2 | Chk | Cell Cycle |
| ***PF-05212384 (PKI-587)*** | 1197160-78-3 | mTOR,PI3K | PI3K/Akt/mTOR |
| ***DCC-2036 (Rebastinib)*** | 1020172-07-9 | Bcr-Abl | Angiogenesis |
| ***CCT128930*** | 885499-61-6 | Akt | PI3K/Akt/mTOR |
| ***A66*** | 1166227-08-2 | PI3K | PI3K/Akt/mTOR |
| ***NU7441 (KU-57788)*** | 503468-95-9 | DNA-PK,PI3K | DNA Damage |
| ***GSK2126458 (GSK458)*** | 1086062-66-9 | PI3K,mTOR | PI3K/Akt/mTOR |
| ***WYE-125132 (WYE-132)*** | 1144068-46-1 | mTOR | PI3K/Akt/mTOR |
| ***A-674563*** | 552325-73-2 | PKA,CDK,Akt | PI3K/Akt/mTOR |
| ***AS-252424*** | 900515-16-4 | PI3K | PI3K/Akt/mTOR |
| ***PF-00562271*** | 939791-38-5 | FAK | Angiogenesis |
| ***Trametinib (GSK1120212)*** | 871700-17-3 | MEK | MAPK |
| ***Flavopiridol HCl*** | 131740-09-5 | CDK | Cell Cycle |
| ***Ibrutinib (PCI-32765)*** | 936563-96-1 | BTK | Angiogenesis |
| ***AS-604850*** | 648449-76-7 | PI3K | PI3K/Akt/mTOR |
| ***CAY10505*** | 1218777-13-9 | PI3K | PI3K/Akt/mTOR |
| ***CHIR-124*** | 405168-58-3 | Chk | Cell Cycle |
| ***NVP-BSK805 2HCl*** | 1092499-93-8(freebase) | JAK | JAK/STAT |
| ***R547*** | 741713-40-6 | CDK | Cell Cycle |
| ***WAY-600*** | 1062159-35-6 | mTOR | PI3K/Akt/mTOR |
| ***TG101209*** | 936091-14-4 | JAK,FLT3,c-RET | JAK/STAT |
| ***GDC-0980 (RG7422)*** | 1032754-93-0 | mTOR,PI3K | PI3K/Akt/mTOR |
| ***A-769662*** | 844499-71-4 | AMPK | PI3K/Akt/mTOR |
| ***CH5132799*** | 1007207-67-1 | mTOR,PI3K | PI3K/Akt/mTOR |
| ***KX2-391*** | 897016-82-9 | Src | Angiogenesis |
| ***GSK1838705A*** | 1116235-97-2 | IGF-1R,ALK | Protein Tyrosine Kinase |
| ***TAK-901*** | 934541-31-8 | Aurora Kinase | Cell Cycle |
| ***AMG-900*** | 945595-80-2 | Aurora Kinase | Cell Cycle |
| ***ZM 336372*** | 208260-29-1 | Raf | MAPK |
| ***PH-797804*** | 586379-66-0 | p38 MAPK | MAPK |
| ***Dacomitinib (PF299804, PF299)*** | 1110813-31-4 | EGFR | Protein Tyrosine Kinase |
| ***AG-1478 (Tyrphostin AG-1478)*** | 153436-53-4 | EGFR | Protein Tyrosine Kinase |
| ***SB415286*** | 264218-23-7 | GSK-3 | PI3K/Akt/mTOR |
| ***Crenolanib (CP-868596)*** | 670220-88-9 | PDGFR | Protein Tyrosine Kinase |
| ***MK-8776 (SCH 900776)*** | 891494-63-6 | CDK,Chk | Cell Cycle |
| ***TG101348 (SAR302503)*** | 936091-26-8 | JAK | JAK/STAT |
| ***GSK1070916*** | 942918-07-2 | Aurora Kinase | Cell Cycle |
| ***PHA-767491*** | 845714-00-3 | CDK | Cell Cycle |
| ***PF-04691502*** | 1013101-36-4 | Akt,mTOR,PI3K | PI3K/Akt/mTOR |
| ***CCT137690*** | 1095382-05-0 | Aurora Kinase | Cell Cycle |
| ***CHIR-98014*** | 252935-94-7 | GSK-3 | PI3K/Akt/mTOR |
| ***AZ 628*** | 878739-06-1 | Raf | MAPK |
| ***AMG-458*** | 913376-83-7 | c-Met | Protein Tyrosine Kinase |
| ***BGT226 (NVP-BGT226)*** | 1245537-68-1 | PI3K,mTOR | PI3K/Akt/mTOR |
| ***Milciclib (PHA-848125)*** | 802539-81-7 | CDK | Cell Cycle |
| ***HER2-Inhibitor-1*** | 937265-83-3 | HER2,EGFR | Protein Tyrosine Kinase |
| ***Varlitinib*** | 845272-21-1 | EGFR | Protein Tyrosine Kinase |
| ***Wortmannin*** | 19545-26-7 | Autophagy,ATM/ATR,PI3K | PI3K/Akt/mTOR |
| ***Fimepinostat*** | 1339928-25-4 | PI3K,HDAC | Cytoskeletal Signaling |
| ***NVP-BVU972*** | 1185763-69-2 | c-Met | Protein Tyrosine Kinase |
| ***Alectinib (CH5424802)*** | 1256580-46-7 | ALK | Protein Tyrosine Kinase |
| ***3-Methyladenine*** | 5142-23-4 | Autophagy,PI3K | PI3K/Akt/mTOR |
| ***Dinaciclib (SCH727965)*** | 779353-01-4 | CDK | Cell Cycle |
| ***Dovitinib (TKI-258) Dilactic Acid*** | 852433-84-2 | PDGFR,FGFR,c-Kit,FLT3,VEGFR | Angiogenesis |
| ***MK-5108 (VX-689)*** | 1010085-13-8 | Aurora Kinase | Cell Cycle |
| ***MK-2461*** | 917879-39-1 | c-Met,PDGFR,FGFR | Protein Tyrosine Kinase |
| ***AZD2014*** | 1009298-59-2 | mTOR | PI3K/Akt/mTOR |
| ***TAK-285*** | 871026-44-7 | EGFR,HER2 | Protein Tyrosine Kinase |
| ***INCB28060*** | 1029712-80-8 | c-Met | Protein Tyrosine Kinase |
| ***Tofacitinib (CP-690550,Tasocitinib)*** | 477600-75-2 | JAK | JAK/STAT |
| ***Sotrastaurin*** | 425637-18-9 | PKC | TGF-beta/Smad |
| ***WP1066*** | 857064-38-1 | JAK | JAK/STAT |
| ***AZD4547*** | 1035270-39-3 | FGFR | Angiogenesis |
| ***CEP-33779*** | 1257704-57-6 | JAK | JAK/STAT |
| ***Dabrafenib (GSK2118436)*** | 1195765-45-7 | Raf | MAPK |
| ***GDC-0068*** | 1001264-89-6 | Akt | PI3K/Akt/mTOR |
| ***INK 128 (MLN0128)*** | 1224844-38-5 | mTOR | PI3K/Akt/mTOR |
| ***BYL719*** | 1217486-61-7 | PI3K | PI3K/Akt/mTOR |
| ***Tyrphostin AG 879*** | 148741-30-4 | HER2 | Protein Tyrosine Kinase |
| ***Torin 2*** | 1223001-51-1 | ATM/ATR,mTOR | PI3K/Akt/mTOR |
| ***TAE226 (NVP-TAE226)*** | 761437-28-9 | FAK | Angiogenesis |
| ***Tideglusib*** | 865854-05-3 | GSK-3 | PI3K/Akt/mTOR |
| ***TPCA-1*** | 507475-17-4 | IκB/IKK | NF-κB |
| ***Torin 1*** | 1222998-36-8 | Autophagy,mTOR | PI3K/Akt/mTOR |
| ***SAR131675*** | 1433953-83-3 | VEGFR | Protein Tyrosine Kinase |
| ***BI-D1870*** | 501437-28-1 | S6 Kinase | PI3K/Akt/mTOR |
| ***Semaxanib (SU5416)*** | 194413-58-6 | VEGFR | Protein Tyrosine Kinase |
| ***Golvatinib (E7050)*** | 928037-13-2 | VEGFR,c-Met | Protein Tyrosine Kinase |
| ***IMD 0354*** | 978-62-1 | IκB/IKK | NF-κB |
| ***WHI-P154*** | 211555-04-3 | JAK,EGFR | JAK/STAT |
| ***TG100713*** | 925705-73-3 | PI3K | PI3K/Akt/mTOR |
| ***GW5074*** | 220904-83-6 | Raf | MAPK |
| ***IKK-16 (IKK Inhibitor VII)*** | 873225-46-8 | IκB/IKK | NF-κB |
| ***PF-562271*** | 717907-75-0 | FAK | Angiogenesis |
| ***NU7026*** | 154447-35-5 | DNA-PK | DNA Damage |
| ***Tyrphostin 9*** | 10537-47-0 | EGFR | Protein Tyrosine Kinase |
| ***ZM 323881 HCl*** | 193000-39-4 | VEGFR | Protein Tyrosine Kinase |
| ***ZM 306416*** | 690206-97-4 | VEGFR | Protein Tyrosine Kinase |
| ***GNF-2*** | 778270-11-4 | Bcr-Abl | Angiogenesis |
| ***S-Ruxolitinib (INCB018424)*** | 941685-37-6 | JAK | JAK/STAT |
| ***PF-477736*** | 952021-60-2 | Chk | Cell Cycle |
| ***Go 6983*** | 133053-19-7 | PKC | TGF-beta/Smad |
| ***BAY 11-7082*** | 19542-67-7 | IκB/IKK,E2 conjugating | NF-κB |
| ***Icotinib*** | 610798-31-7 | EGFR | Protein Tyrosine Kinase |
| ***CHIR-99021 (CT99021) HCl*** | 252917-06-9(freebase) | GSK-3 | PI3K/Akt/mTOR |
| ***TAK-715*** | 303162-79-0 | p38 MAPK | MAPK |
| ***Pazopanib*** | 444731-52-6 | PDGFR,c-Kit,VEGFR | Protein Tyrosine Kinase |
| ***Piceatannol*** | 10083-24-6 | Syk | Angiogenesis |
| ***SC-514*** | 354812-17-2 | IκB/IKK | NF-κB |
| ***Tofacitinib (CP-690550) Citrate*** | 540737-29-9 | JAK | JAK/STAT |
| ***Fingolimod (FTY720) HCl*** | 162359-56-0 | S1P Receptor | GPCR & G Protein |
| ***VX-702*** | 745833-23-2 | p38 MAPK | MAPK |
| ***AP26113*** | 1197958-12-5 | ALK | Protein Tyrosine Kinase |
| ***MEK162 (ARRY-162, ARRY-438162)*** | 606143-89-9 | MEK | MAPK |
| ***PP2*** | 172889-27-9 | Src | Angiogenesis |
| ***CZC24832*** | 1159824-67-5 | PI3K | PI3K/Akt/mTOR |
| ***IPI-145 (INK1197)*** | 1201438-56-3 | PI3K | Angiogenesis |
| ***XL388*** | 1251156-08-7 | mTOR | PI3K/Akt/mTOR |
| ***XL019*** | 945755-56-6 | JAK | JAK/STAT |
| ***PD168393*** | 194423-15-9 | EGFR | Protein Tyrosine Kinase |
| ***AZ20*** | 1233339-22-4 | ATM/ATR | PI3K/Akt/mTOR |
| ***PP1*** | 172889-26-8 | Src | Angiogenesis |
| ***MK-8745*** | 885325-71-3 | Aurora Kinase | Cell Cycle |
| ***LDK378*** | 1032900-25-6 | ALK | Protein Tyrosine Kinase |
| ***IPA-3*** | 42521-82-4 | PAK | Cytoskeletal Signaling |
| ***VE-822*** | 1232416-25-9 | ATM/ATR | PI3K/Akt/mTOR |
| ***AZD3463*** | 1356962-20-3 | ALK | Protein Tyrosine Kinase |
| ***NU6027*** | 220036-08-8 | CDK | Cell Cycle |
| ***TIC10*** | 41276-02-2 | Akt | PI3K/Akt/mTOR |
| ***CGK 733*** | 905973-89-9 | ATM/ATR | DNA Damage |
| ***AZD1080*** | 612487-72-6 | GSK-3 | PI3K/Akt/mTOR |
| ***10058-F4*** | 403811-55-2 | c-Myc | Cell Cycle |
| ***LY2835219*** | 1231930-82-7 | CDK | Cell Cycle |
| ***SSR128129E*** | 848318-25-2 | FGFR | Angiogenesis |
| ***AVL-292*** | 1202757-89-8 | BTK | Angiogenesis |
| ***SKI II*** | 312636-16-1 | S1P Receptor | GPCR & G Protein |
| ***GZD824*** | 1421783-64-3 | Bcr-Abl | Angiogenesis |
| ***RKI-1447*** | 1342278-01-6 | ROCK | Cell Cycle |
| ***BIO*** | 667463-62-9 | GSK-3 | PI3K/Akt/mTOR |
| ***Ro 31-8220 Mesylate*** | 138489-18-6 | PKC | TGF-beta/Smad |
| ***Skepinone-L*** | 1221485-83-1 | p38 MAPK | MAPK |
| ***AZD2858*** | 486424-20-8 | GSK-3 | PI3K/Akt/mTOR |
| ***CNX-774*** | 1202759-32-7 | BTK | Angiogenesis |
| ***CO-1686 (AVL-301)*** | 1374640-70-6 | EGFR | Protein Tyrosine Kinase |
| ***TAK-632*** | 1228591-30-7 | Raf | MAPK |
| ***ZCL278*** | 587841-73-4 | Rac | Cell Cycle |
| ***WZ4003*** | 1214265-58-3 | AMPK | PI3K/Akt/mTOR |
| ***EHop-016*** | 1380432-32-5 | Rac | Cell Cycle |
| ***TG003*** | 300801-52-9 | CDK | Cell Cycle |
| ***Sorafenib*** | 284461-73-0 | Raf | MAPK |
| ***AR-A014418*** | 487021-52-3 | GSK-3 | PI3K/Akt/mTOR |
| ***GSK2636771*** | 1372540-25-4 | PI3K | PI3K/Akt/mTOR |
| ***PQ 401*** | 196868-63-0 | IGF-1R | Protein Tyrosine Kinase |
| ***ZM 39923 HCl*** | 1021868-92-7 | JAK | JAK/STAT |
| ***SMI-4a*** | 438190-29-5 | Pim | JAK/STAT |
| ***VE-821*** | 1232410-49-9 | ATM/ATR | DNA Damage |
| ***AG-18*** | 118409-57-7 | EGFR | Protein Tyrosine Kinase |
| ***CEP-32496*** | 1188910-76-0 | CSF-1R,Raf | MAPK |
| ***AZD5363*** | 1143532-39-1 | Akt | PI3K/Akt/mTOR |
| ***TCS 359*** | 301305-73-7 | FLT3 | Angiogenesis |
| ***Tyrphostin AG 1296*** | 146535-11-7 | FGFR,c-Kit,PDGFR | Protein Tyrosine Kinase |
| ***NSC 23766*** | 1177865-17-6 | Rac | Cell Cycle |
| ***PRT062607 (P505-15, BIIB057) HCl*** | 1370261-97-4 | Syk | Angiogenesis |
| ***Butein*** | 487-52-5 | EGFR | Protein Tyrosine Kinase |
| ***GDC-0349*** | 1207360-89-1 | mTOR | PI3K/Akt/mTOR |
| ***BMS-345541*** | 445430-58-0 | IκB/IKK | NF-κB |
| ***ETP-46464*** | 1345675-02-6 | mTOR,ATM/ATR | PI3K/Akt/mTOR |
| ***Pacritinib (SB1518)*** | 937272-79-2 | JAK | JAK/STAT |
| ***P276-00*** | 920113-03-7 | CDK | Cell Cycle |
| ***Bardoxolone Methyl*** | 218600-53-4 | IκB/IKK | NF-κB |

**Supplemental Table 1: Drugs screened in this study**

Spreadsheet listing drugs, CAS number, drug target and pathway for the drugs screened in this study.

**
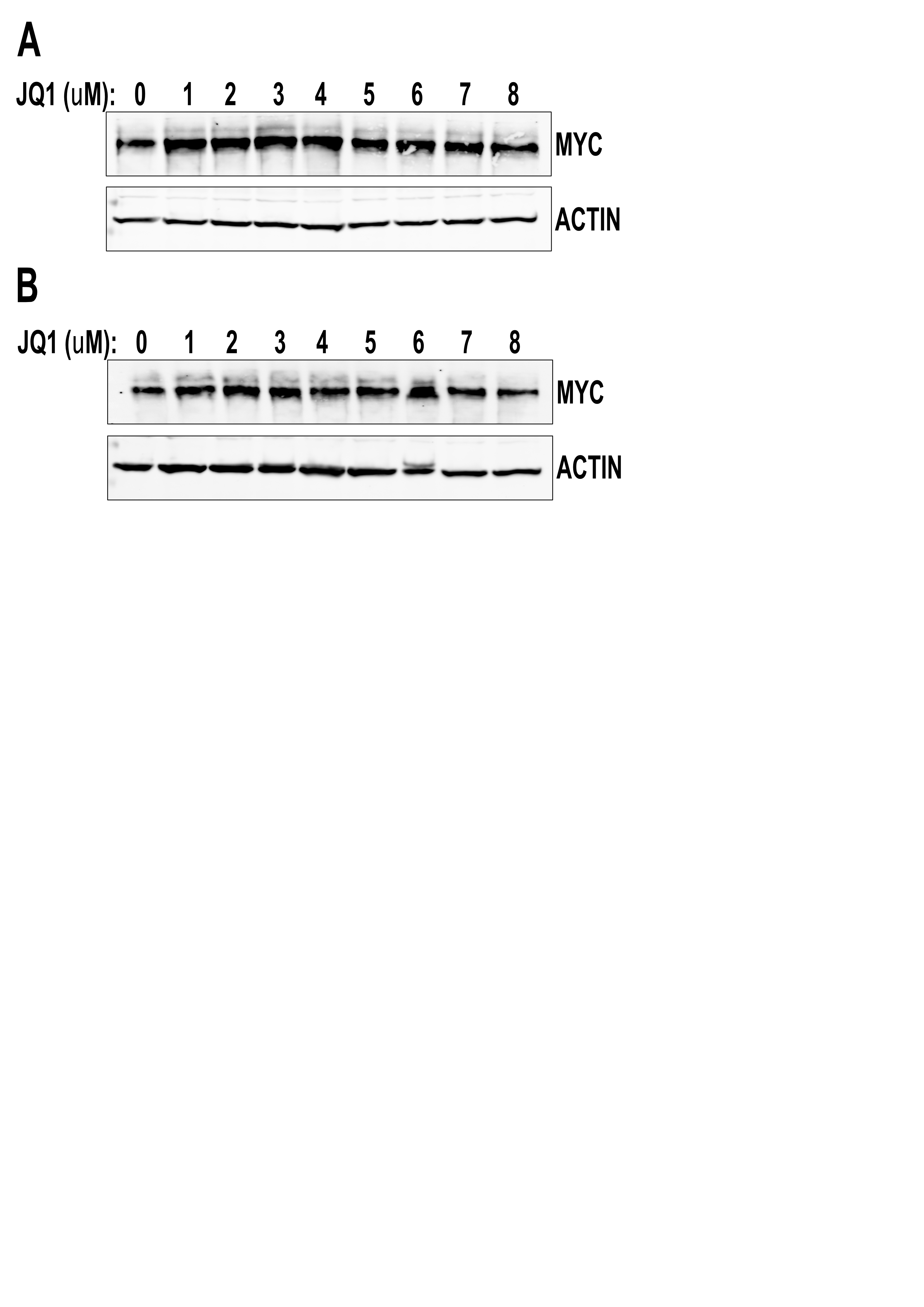
**

**Supplemental Figure 1: JQ1 has no effect on MYC expression in platinum resistant mouse SCLC cell lines.**

**(A)** B37R and **(B)** EN84R cells were treated with the indicated doses of JQ1 and MYC expression determined by western blot. Blots were re-probed with an actin antibody as a loading control. Data is representative of three independent experiments.
